# A Novel Mutation in Brain Tumor Causes Both Neural Over-Proliferation and Neurodegeneration in Adult *Drosophila*

**DOI:** 10.1534/g3.118.200627

**Published:** 2018-08-20

**Authors:** Carin Loewen, Grace Boekhoff-Falk, Barry Ganetzky, Stanislava Chtarbanova

**Affiliations:** * Laboratory of Genetics, University of Wisconsin-Madison, Madison, WI 53706; † Department of Cell and Regenerative Biology, School of Medicine and Public Health, University of Wisconsin-Madison, Madison, WI 53705; ‡ Department of Biological Sciences, University of Alabama, Tuscaloosa, AL 35487

**Keywords:** Drosophila, brain tumor, neurodegeneration, cell proliferation, prolyl4-hydroxylase

## Abstract

A screen for neuroprotective genes in *Drosophila melanogaster* led to the identification of a mutation that causes extreme, progressive loss of adult brain neuropil in conjunction with massive brain overgrowth. We mapped the mutation to the *brain tumor* (*brat*) locus, which encodes a tripartite motif-NCL-1, HT2A, and LIN-41 (TRIM-NHL) RNA-binding protein with established roles limiting stem cell proliferation in developing brain and ovary. However, a neuroprotective role for *brat* in the adult *Drosophila* brain has not been described previously. The new allele, *brat^cheesehead^* (*brat^chs^*), carries a mutation in the coiled-coil domain of the TRIM motif, and is temperature-sensitive. We demonstrate that mRNA and protein levels of neural stem cell genes are increased in heads of adult *brat^chs^* mutants and that the over-proliferation phenotype initiates prior to adult eclosion. We also report that disruption of an uncharacterized gene coding for a presumptive prolyl-4-hydroxylase strongly enhances the over-proliferation and neurodegeneration phenotypes. Together, our results reveal an unexpected role for *brat* that could be relevant to human cancer and neurodegenerative diseases.

Neurodegenerative diseases such as Alzheimer's disease and Parkinson's disease affect millions of people worldwide and are leading causes of death in the United States (https://www.cdc.gov/nchs/fastats/deaths.htm). They are characterized by progressive loss of neuronal tissue and currently are untreatable. Although substantial progress has been made in understanding the cellular and molecular basis of these disorders, a complete understanding of the mechanisms underlying neurodegeneration still is lacking and effective therapies for human neurodegenerative diseases are limited. Here, we describe progressive neurodegeneration in mutants for a well-studied neural development gene and propose that pathways regulated by homologs of this gene may be relevant to human neurodegeneration.

*Drosophila melanogaster* is a powerful model organism for investigating molecular and cellular mechanisms that underlie neurodegeneration, and many genes and pathways with roles in neuroprotection and neurodegeneration have been identified and characterized in this organism (Bilen and Bonini 2005; Lessing and Bonini 2009). For example, ATPase-α was first shown to play a neuroprotective role in *Drosophila* (Palladino *et al.* 2003) and subsequently identified as a neuroprotective gene in humans (De Carvalho Aguiar *et al.* 2004). Mutation in the Swiss cheese protein was first shown to cause neurodegeneration in *Drosophila* (Kretzschmar *et al.* 1997); and later, mutations in the human ortholog of Swiss cheese, PNPLA6, were shown to cause motor neuron disease characterized by axonal degeneration (Hein *et al.* 2010), as well as some forms of blindness due to photoreceptor degeneration (Kmoch *et al.* 2015). Flies also have provided insights into disease mechanisms, including the processing of amyloid precursor protein (APP) and neurotoxicity Aβ42 in Alzheimer’s Disease (Loewer *et al.* 2004), the interaction of *parkin* and *Pink1* at mitochondria (Greene *et al.* 2003; Clark *et al.* 2006) and the spreading of Huntingtin aggregates and subsequent neuronal death (Babcock and Ganetzky 2015), as well as various downstream mechanisms of neurotoxicity in tauopathy (Khurana *et al.* 2006; Dias-Santagata *et al.* 2007; Fulga *et al.* 2007; Loewen and Feany 2010; Frost *et al.* 2014; Frost *et al.* 2016). Studies of other mutants in *Drosophila*, have also revealed that sustained activation of the innate immune response in neurons and glia leads to progressive neurodegeneration (Chinchore *et al.* 2012; Petersen *et al.* 2012; Cao *et al.* 2013; Kounatidis *et al.* 2017).

The *Drosophila brain tumor* (*brat*) gene plays an essential role in asymmetric cell division of neural stem cells (neuroblasts) and its function during this process in larval development has been investigated extensively (Bello *et al.* 2006; Betschinger *et al.* 2006; Lee *et al.* 2006). *brat* encodes a conserved TRIM-NHL (tripartite motif-NCL-1, HT2A, and LIN-41) RNA-binding protein (Arama *et al.* 2000). In addition to its role in neuroblast division, Brat also is a translational repressor of mRNAs including *hunchback* (*hb*) during embryonic patterning (Sonoda and Wharton 2001; Loedige *et al.* 2014; Laver *et al.* 2015). The N-terminal TRIM domain of Brat consists of two B-boxes and a coiled-coil domain, but lacks the RING domain found in most TRIM proteins (Wulczyn *et al.* 2011). B-boxes are zinc finger domains implicated in protein-protein interactions, substrate recognition, and interaction with RNA polymerase II (Crocco and Botto 2013), while coiled-coil domains mediate protein-protein interactions, including multimerization (Lupas 1996; Reymond *et al.* 2001; Grigoryan and Keating 2008). The C-terminal NHL domain has multiple functions, including binding to mRNA to regulate translation (Loedige *et al.* 2014; Loedige *et al.* 2015), binding to other RNA regulatory proteins (Sonoda and Wharton 2001), and binding to Miranda for partitioning during asymmetric cell division (Lee *et al.* 2006). Additional roles for Brat in *Drosophila* include regulation of germline stem cell differentiation in the ovary (Harris *et al.* 2011; Newton *et al.* 2015), and regulation of synaptic endocytosis at the fly neuromuscular junction (NMJ) (Shi *et al.* 2013). Moreover, reduction of *brat* expression specifically in the *Drosophila* mushroom body (a structure central to learning and memory) leads to axonal retraction (Marchetti *et al.* 2014), indicating that Brat plays a role in the maintenance of neuronal integrity. TRIM-NHL proteins are evolutionarily conserved, and alterations in mammalian orthologs of *brat* with predominant brain expression have been associated with neuropathology or cancer (Tocchini and Ciosk 2015). Consistent with a neuroprotective role, mutations in *TRIM2* were linked to Alzheimer’s disease (Schonrock *et al.* 2012) and axonal neuropathy (Ylikallio *et al.* 2013). On the other hand, deletions of *TRIM3* are frequently found in primary human gliomas pointing to a tumor suppressor role for TRIM3 (Boulay *et al.* 2009).

Here, we characterize a novel *Drosophila* mutant, *cheesehead (chs)* that exhibits both aberrant continued proliferation of cells in the adult brain and progressive neurodegeneration. Furthermore, we identify *chs* as a temperature-sensitive allele of *brat (brat^chs^)*, in which a point mutation leads to an amino acid change in the coiled-coil domain of the protein. Thus, we find an unexpected role for Brat in neurodegeneration that is intimately linked to neural hypertrophy. Finally, we report that the dual phenotype of *brat^chs^* flies is enhanced by a mutation in a putative prolyl-4 hydroxylase-coding gene. This represents a previously unknown interaction for Brat that may reveal a new pathway in which Brat functions that could be relevant to human neurodegenerative and neoplastic diseases.

## Materials and Methods

### *Drosophila* stocks and reagents

Flies were maintained on cornmeal-molasses medium at 25° unless otherwise stated. The collection of ENU-mutagenized *Drosophila*, including line 867 was a kind gift of Dr. Steven Robinow (University of Hawaii). *UAS-brat*, *brat^ts1^*, *brat^fs1^ and brat^k06028^* were obtained from Dr. Cheng-Yu Lee (University of Michigan). *R9D11-mCD8-GFP* was obtained from Dr. Jill Wildonger (University of Wisconsin-Madison). The following fly lines were obtained from the Bloomington Drosophila Stock Center at Indiana University: *Df(2L)ED1272* (#24116), *Df(2L)ED1203* (#8935), *Df(2L)BSC341* (#24365), *Df(2L)ED1231* (#9174), *pcna-GFP* (#25749), *worniu-Gal4* (#56554), *nSyb-Gal4* (#51635), *CG15864^MB04166^* (#24678), *UAS-NICD* (#52008), *OK107-Gal4* (#854), *UAS-mCD8-GFP* (#5137), and *insc-Gal4* (#8751). *UAS-Brat-RNAi* (#105054) was obtained from the Vienna Drosophila Resource Center (Dietzl *et al.* 2007).

### Histology

Histological analysis was done as previously described (Cao *et al.* 2013). Fly heads were severed and placed in fresh Carnoy’s fixative (ethanol: chloroform: glacial acetic acid in the ratio 6:3:1) overnight at 4°. Heads were then transferred in 70% ethanol and processed into paraffin using standard histological procedures. Embedded heads were sectioned at 5μm, and stained with hematoxylin and eosin. Images were taken using the 20X objective of a Nikon light microscope (Nikon, Japan), equipped with a QImaging camera and images were generated using QImaging software (QImaging company, Canada) and processed with Photoshop CS5.

### DNA-sequencing

DNA from a single fly was isolated as previously described (Gloor *et al.* 1993) and PCR reactions carried out to amplify all the exon-coding regions of the *brat* gene. Another mutagenized line from the same collection was used as a background control strain. PCR products were gel-purified using the Wizard SV Gel and PCR Clean-Up System from Promega (#A9282) according to manufacturer’s instructions. Sequencing reactions were carried out using Big Dye Terminator v3.1 Cycle Sequencing Kit from Applied Biosystems (#4337455). Subsequently samples were beads-purified and sent to the University of Wisconsin Biotechnology Center DNA sequencing Facility (425 Henry Mall, Madison WI-53706). Schematic representation of the region amplified for sequencing analysis and the sequences of primers used are shown in Figure S1. Splinkerette PCR (spPCR) for the mapping of the *PCNA-GFP* insertion was performed as previously described (Potter and Luo 2010).

### Immunohistochemistry

Brains were dissected in PBS1X and fixed in 4% formaldehyde in PBS1X for 20-45 min at room temperature (RT). Brains were then placed in blocking solution (PBS1X with 0.1% Triton-X100 and 0.1% normal goat serum) for 2 hr at room temperature. Brains were then incubated in primary antibodies diluted in blocking solution. Next, brains were washed 5X in PBS1X and then incubated in secondary antibodies diluted in blocking solution for 2 hr at RT. Finally, brains were washed 5X in PBS1X and mounted in Vectashield (Vector Laboratories, Burlingame, CA).

The following monoclonal antibodies were obtained from the Developmental Studies Hybridoma Bank, created by the NICHD of the NIH and maintained at The University of Iowa, Department of Biology, Iowa City, IA 52242: mouse anti-Prospero (MR1A, contributed by C. Q. Doe, University of Oregon, used at 3:1 at 4° for 2 days), mouse anti-Repo (8D12, contributed by C. Goodman, University of California-Berkley, used at 1:50 at 4° overnight), rat anti-Elav (7E8A10, contributed by G.E. Rubin, Janelia Farm, used at 1:100 at 4° overnight) and mouse anti-Fasciclin II (anti-Fas II) (1D4, contributed by C. Goodman, University of California-Berkley, used at 1:15 at 4° for 3 days). Rabbit anti-cleaved-Dcp1 (9578S, Cell Signaling Technology, used at 1:100 at 4° for 2 days), chicken anti-GFP (Cat # A10262, Invitrogen, used at 1:1000 at 4° overnight), mouse anti-Asense (gift from Cheng-Yu Lee, University of Michigan, used at 1:400 for 3 hr at room temperature), and rabbit anti-PH3 (sc-8656-R, Santa Cruz Biotechnology, used at 1:1000, at 4° overnight). Secondary antibodies from Invitrogen were used at 1:200 at room temperature for 2 hr: anti-mouse Alexa Fluor 568 (#A11031), anti-chicken Alexa Fluor 488 (#A11039w), anti-mouse Alexa Fluor 405 (#A31553), anti-rabbit Alexa Fluor 568 (#A11036), anti-rat Alexa Fluor 633 (#A21094).

### Gene expression

Quantitative Real-Time PCR (qPCR) was used to measure mRNA expression. 5 to15 fly heads of the indicated genotypes were severed and RNA was isolated using TrizolRT (Molecular Research center, Inc. Cincinnati, OH, USA) according to the manufacturer instructions. cDNA was synthesized using an iScript cDNA Synthesis Kit (Bio-Rad, Hercules, CA, USA). Real-time PCR was carried out by using iQ SYBR Green Supermix (Bio-Rad, Hercules, CA, USA) according to the manufacturer instructions. The sequences of the primers used are as follows (5′-3′):

*asense:* forward (CAGTGATCTCCTGCCTAGTTTG), reverse (GTGTTGGTTCCTGGTATTCTGATG);
*deadpan*: forward (CGCTATGTAAGCCAAATGGATGG), reverse (CTATTGGCACACTGGTTAAGATGG);
*miranda*: forward (CCCAATTGGAGCTGGACAACA), reverse (GGTGTTCCCAGCAGAGAGG);
*klumpfuss*: forward (CAGAGCAATCTGCCCCAAGA), reverse (TGGTGTGCAGGTAATAGCCG);
*pointed RC*, *RE*: forward (CGACTGCGAACAATCTGGTG), reverse AGTTGACATCCGAGTCCGTG;
*earmuff*: forward (GGATCCATCGAGGACAGCAG), reverse (GAGGTTGTAGTGGGCGTTGA);
*earmuff* set 2: forward (TCAACGCCCACTACAACCTC), reverse (CCGCAGACCTTGCAAACAAA);
*earmuff* set 3: forward (GGGACTTGAGCGCATTTTTC), reverse (TTCTTGTCGTTGTGCGTGTG) (Eroglu *et al.* 2014);
*brat*: forward (GTGGTTAGTGGCGCTGGAG), reverse (GGATAGATAGTGGCCGAAAGC).

### Statistical analysis

Data were analyzed using GraphPad Prism 5 software (GraphPad Software, Inc., La Jolla, CA). Significance of gene expression was analyzed using a non-parametric Mann Whitney *U*-test. Comparison of three or more samples was done using one-way ANOVA. A Chi-square test was used to compare differences in brains exhibiting over-proliferation and neurodegeneration. In all tests, *P* < 0.05 was considered significant.

### Data availability

File S1 contains primer sequences used for amplification and sequencing of *brat* coding region. *Drosophila* strains and reagents are available upon request. Supplemental material available at Figshare: https://doi.org/10.25387/g3.6981743.

## Results

### A mutation in the coiled-coil domain in *brat^chs^* leads to both neural over-proliferation and neurodegeneration

**Figure 1:**
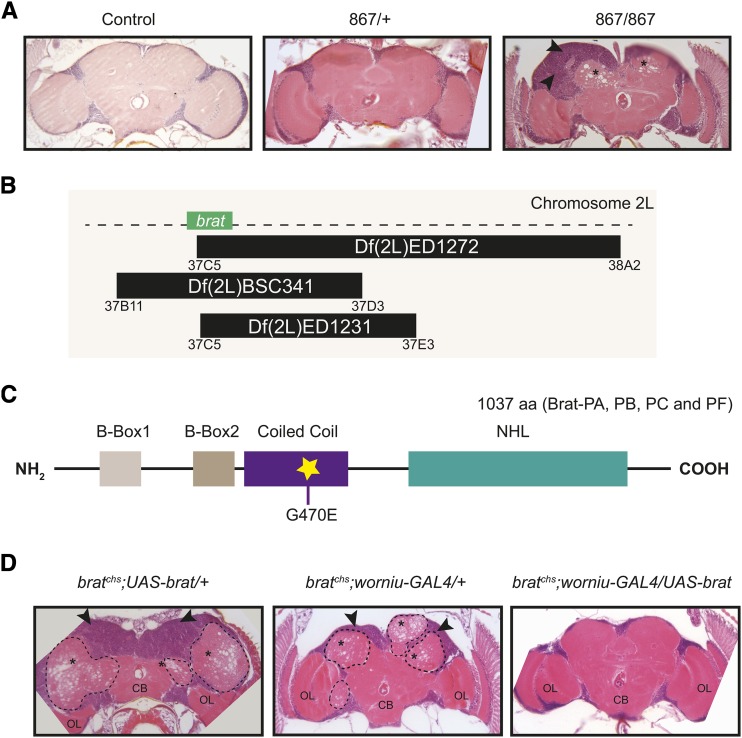
Phenotypic and molecular characterization of *cheesehead*, a novel allele of *brat*. A. Histological analysis of adult brains from mutant line *867*. Representative 5-μm paraffin sections at approximately midbrain show neurodegeneration (asterisks) and cellular proliferation (arrowheads) in 18-20 day homozygous 867 (n = 4), but not control (*w^1118^*; n = 10) or heterozygous 867 (n = 19) age-matched flies. B. Schematic representation of three of the deficiency lines used to map the mutation in line 867 to the *brat* locus. C. Schematic representation of the Brat protein showing the location and nature of the amino acid change in the Coiled-Coil domain in *brat^chs^*. The polypeptide corresponding to Brat-PA, PB, PC and PF isoforms is presented in the figure. D. Representative 5-μm paraffin sections at approximately midbrain of 4-11 day *brat^chs^*; *UAS-brat/+* (n = 2), *brat^chs^*; *worniu-Gal4/+* (n = 7) and *brat^chs^*; *worniu-Gal4 > UAS-brat* (n = 12) flies raised and aged at 29°C. Both neurodegeneration (asterisks) and cellular overgrowth (arrowheads) are rescued when *brat* cDNA is expressed in *brat^chs^* neuroblasts. ∼30% (n = 7) of the homozygous *brat^chs^* flies carrying the *worniu-Gal4* driver alone exhibited partial rescue, suggesting the presence of a weak suppressor of *brat^chs^* located on the *worniu-Gal4* chromosome (data not shown). Ectopic neuropil is outlined with dotted lines. The central brain (CB) and optic lobes (OL) are present, but displaced in the mutants.

We performed a direct histological screen for *Drosophila* mutants exhibiting neurodegeneration of 81 lines that showed climbing defects from a larger collection of 310 ENU-mutagenized lines by examining hematoxylin and eosin-stained brain sections from each line and scoring for holes in brain tissue. This type of spongiform pathology of *Drosophila* brain tissue has previously been correlated with both neuronal and glial cell death (*e.g.*, (Kretzschmar *et al.* 1997) and will be referred as neurodegeneration thereafter. We identified line *867* as harboring a recessive mutation that leads to degeneration in the adult brain neuropil (regions surrounding asterisks in Figure 1A). Surprisingly, in addition to neurodegeneration, we also observed an increase in the number of cell bodies at the brain surface (regions indicated by arrowheads in Figure 1A), and the appearance of extra neuropil undergoing degeneration as indicated by its spongiform appearance (visible in area enclosed by dotted lines in Figure 1D). Although *867* brains are overgrown, distinct brain regions, such as the optic lobes (OL in Figure 1D) and the central brain (CB in Figure 1D), seem appropriately formed, but sometimes are displaced by extra neuropil. Additionally, we cannot rule out the possibility that there are defects in specific brain regions that we did not detect in our histological preparations.

We named this mutation, *cheesehead* (*chs*), referring to the numerous holes present in the neuropil. Using recombination and deficiency mapping (see below) together with DNA sequence analysis, we mapped the neurodegeneration-causing mutation to the previously identified *brain tumor* (*brat*) gene, and thus designate our new allele as *brat^chs^*. Three deficiency lines (black bars in Figure 1B) failed to complement *brat^chs^*. These results indicated that the mutation is on the left arm of the second chromosome in the region of overlap among these deficiencies. This region encompasses approximately 118 genes, including *brat*. Because of the supernumerary cells in the *867* brains, we focused our attention on the *brat* locus. DNA sequence analysis of coding regions in *867* revealed a point mutation in the *brat* locus (Figure S1). This mutation is a G → A nucleotide change at position 37,739 and is predicted to result in a glycine to glutamic acid (G→E) change in the coiled-coil domain of the protein (Figure 1C).

To determine whether neural over-proliferation and neurodegeneration were both caused by *brat^chs^*, we performed rescue experiments with the Gal4/UAS system (Brand and Perrimon 1993). Full-length, wild type *brat* cDNA under control of the UAS element (*UAS-brat*) (Komori *et al.* 2014) was expressed in *Drosophila* neural stem cells (neuroblasts) using a *worniu-Gal4* (*wor-Gal4*) driver in flies that were homozygous for *brat^chs^*. Homozygous *brat^chs^* flies reared at 29° and carrying either *UAS-brat* or the *wor-Gal4* driver alone exhibited both over-proliferation (regions indicated by arrowheads in the left and middle panels of Figure 1D) and neurodegeneration (regions surrounding asterisks in the left and middle panels of Figure 1D). In contrast, 100% (n = 12) of homozygous *brat^chs^* flies carrying both *UAS-brat* and *wor-Gal4* exhibited full rescue of both over-proliferation and neurodegeneration (right panel of Figure 1D). These experiments confirmed that the *brat^chs^* mutation is the cause of the observed phenotypes in *867* brains. Moreover, we were able to recapitulate both the overproliferation and neurodegeneration phenotypes using a *UAS-brat_RNAi_* construct under the control of the neuroblast-specific driver *Insc-Gal4* (Figure S2). Reduced activity of *brat* in neuroblasts leads to increased levels of activated Notch (Mukherjee *et al.* 2016). We therefore tested whether overexpression of the active Notch intracellular domain (NICD) alone is sufficient to recapitulate the neurodegeneration phenotype. Indeed, we observed spongiform pathology in *wor-Gal4 >UAS-NICD* brains, suggesting that deregulated Brat-dependent Notch signaling contributes to neurodegeneration (Figure S2).

### Neurodegeneration in *brat^chs^* is progressive and coincident with activation of caspase Dcp-1

**Figure 2:**
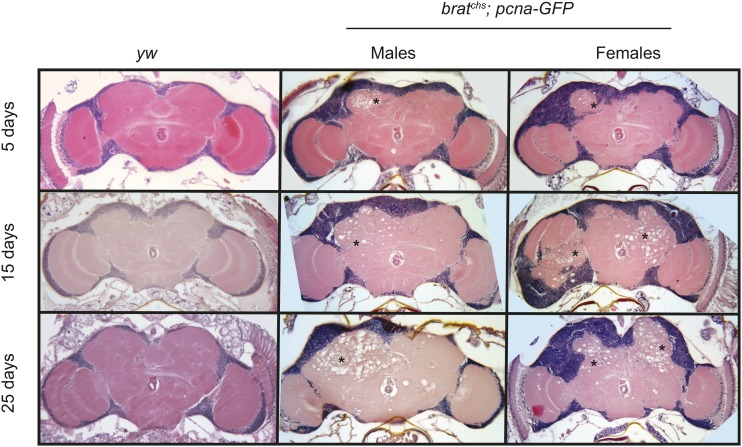
Neurodegeneration in *brat^chs^* is progressive and age dependent. Representative hematoxylin and eosin-stained 5-μm paraffin sections at approximately midbrain of 5, 15 and 25 day *yw* (control) and homozygous *brat^chs^*; *pcna-GFP* flies (*yw*: 5 day, n = 14; 15 day, n = 11; and 25 day, n = 15; *brat^chs^*; *pcna-GFP* females: 5 day, n = 4; 15 day, n = 3; and 25 day, n = 6; *brat^chs^*; *pcna-GFP* males: 5 day, n = 4; 15 day, n = 4; and 25 day, n = 6). Neurodegeneration (asterisks) in both male and female *brat^chs^*; *pcna-GFP* flies, as assessed by the number and density of holes in the neuropil, becomes more severe as the flies age.

To test whether neurodegeneration in *brat^chs^* is progressive and age-dependent, we examined brain sections at 5, 15 and 25 days post-eclosion (Figure 2). For these experiments we used *brat^chs^*; *pcna-GFP* flies (see next section), which are viable at 25° and exhibit both higher penetrance and higher expressivity than *brat^chs^*. At 5 days post-eclosion, *brat^chs^* brains exhibited detectable, but mild, degeneration in both males and females. In contrast, by 15 days post-eclosion, the lesions in the neuropil became larger and more numerous in both sexes (asterisks in Figure 2). This phenotype was even more severe at 25 days post-eclosion (asterisks in Figure 2), indicating that the neurodegeneration observed in *brat^chs^* mutants worsens over time.

**Figure 3:**
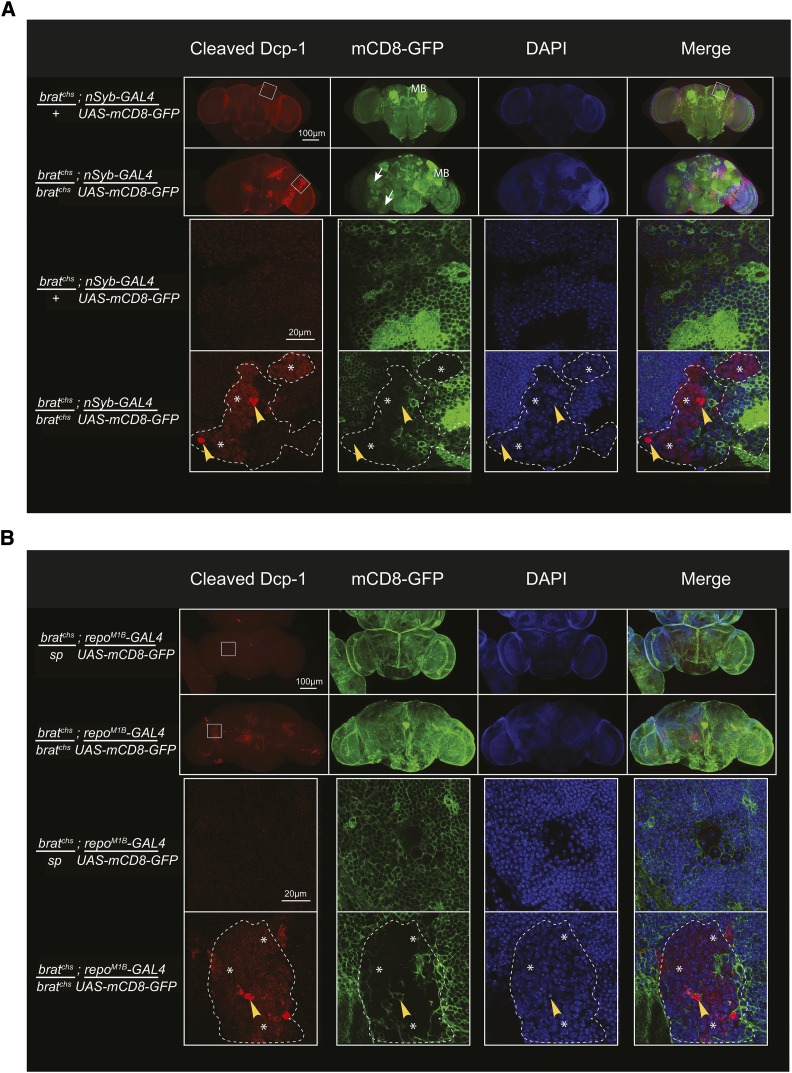
Activation of Dcp-1 in *brat^chs^* brains occurs primarily in non-differentiated cells. A. Confocal microscope images of *brat^chs^/+* (control) and *brat^chs^* brains expressing *mCD8-GFP* under the control of *nSyb-Gal4* and stained for the cleaved caspase Dcp-1 (red) and GFP (green). Nuclei are stained with DAPI (blue). Low magnification images are presented in the top two rows and high magnification images of the zones outlined by the white squares are in the panels of the two lower rows. In *brat^chs^*, Dcp-1-positive cells are present in regions of the brain containing large nuclei (surrounded by the dashed lines) and do not express the neuronal differentiation marker *nSyb*. Arrows indicate the ectopic neuropil present in *brat^chs^* brains. MB: mushroom bodies. B. Confocal microscope images of *brat^chs^/+* (control) and *brat^chs^* brains expressing *mCD8-GFP* under the control of *repo^M1B^-Gal4* and stained for the cleaved caspase Dcp-1 (red) and GFP (green). Nuclei are stained with DAPI (blue). Low magnification images are presented in the panels of the top two rows and high magnification images of the zones outlined by the white squares are in the panels of the two lower rows. In *brat^chs^*, Dcp-1-positive cells are found in regions of the brain containing large nuclei (surrounded by the dashed lines). A subset of the strongly Dcp-1-positive cells expresses the glial marker, *repo* (arrowheads). Asterisks indicate weakly positive Dcp-1 cells.

To examine whether progressive neurodegeneration in *brat^chs^* is associated with an increase in apoptosis, we stained whole brains with anti-cleaved Dcp-1 (Death caspase-1) antibody (Hay and Guo 2006; Florentin and Arama 2012; Sarkissian *et al.* 2014). Dcp-1 is a fly homolog of mammalian Caspase-3, an effector caspase for apoptosis that is activated upon cleavage by initiator caspases (Ryoo and Bergmann 2012). Compared with brains from heterozygous controls, we found substantial staining for cleaved Dcp-1 in *brat^chs^* homozygotes (Figures 3A and B) indicating that apoptosis was activated in mutant brains.

Two classes of anti-cleaved Dcp-1-labeled cells in *brat^chs^* tumors (high magnification images in Figures 3A and B) were observed. The first class is weakly positive for cleaved Dcp-1. These weakly positive cells are found in zones within the tumors and compose about half of each tumor region. The second class of cleaved Dcp-1 positive cells has much stronger staining. These strongly Dcp-1 positive cells were relatively rare and found within the weakly Dcp-1 positive zones (indicated with asterisks in Figure 3A and B). Many strongly labeled cells have undetectable DAPI labeling, whereas others have pyknotic nuclei (arrowhead in the high magnification image in Figure 3B). This is consistent with the strongly Dcp-1 positive cells undergoing death.

To identify the Dcp-1 positive cells, we used neuronal, glial and neural progenitor markers. Specifically, we first expressed a membrane-targeted GFP transgene (*UAS-mCD8-GFP*) using either a neuronal-specific driver (*nSyb-Gal4*) or a glial-specific driver (*Repo^M1B^-Gal4*) (Figure 3). Surprisingly, we found that none of the cells either strongly or weakly positive for cleaved Dcp-1- expressed *nSyb* (Figure 3A) and only a few expressed *repo* (Figure 3B). As described above, we delineated the tumor regions based on the presence of weakly DAPI-stained, large nuclei. The vast majority of Dcp-1-positive cells did not express either *nSyb* or *repo*, and thus, do not appear to be either differentiated neurons or glia.

**Figure 4:**
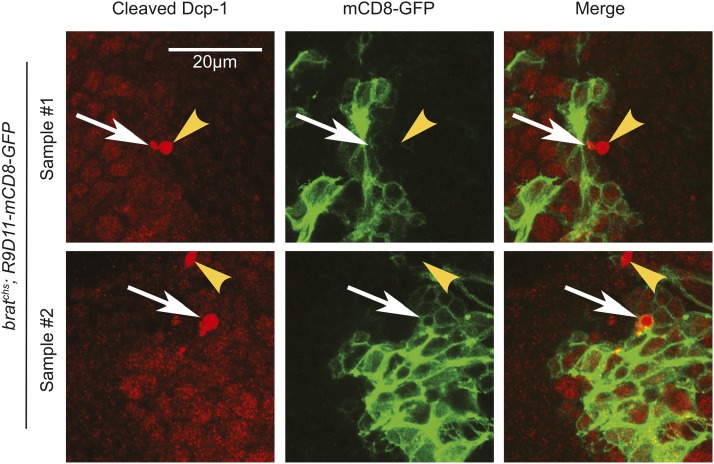
*erm* expression is detected in a subset of cells that are positive for cleaved Dcp-1 in *brat^chs^* brains. Confocal microscope images of *brat^chs^*; *R9D11-mCD8-GFP* brains stained for cleaved caspase Dcp-1 (red) and GFP (green). Some Dcp-1-positive cells also express GFP under control of the *erm* promoter (samples 1 and 2), although not all do (most clear in sample 1). Most of the GFP-positive cells show weak labeling for cleaved Dcp-1. However, some GFP-positive cells have the strong cleaved Dcp-1 signal (arrows). Arrowheads show strong Dcp-1 signals with no GFP expression.

One possibility is that these Dcp-1- positive cells lacking both *nSyb* and *repo* expression were neural progenitors. We tested this idea by determining whether they expressed GFP from the *erm* reporter transgene, *R9D11-mCD8-GFP* (Figure 4). While not all Dcp-1 positive cells expressed R9D11-mCD8-GFP (Figure 4, sample 1), some did (Figure 4, sample 2). The majority of cells that were positive for both R9D11-mDC8-GFP and cleaved Dcp-1 showed only weak Dcp-1 labeling. 35% of strongly Dcp-1 positive cells also expressed R9D11-mCD8-GFP (Figure 4, arrows). However 65% of strongly Dcp-1 positive cells did not express GFP (Figure 4, arrowheads; n = 82 strongly Dcp-1 positive cells from 3 different brains). On the basis of these observations, we conclude that many of the Dcp-1 positive cells are neural progenitor cells.

### Cells continue to proliferate in adult brains in *brat^chs^*

**Figure 5:**
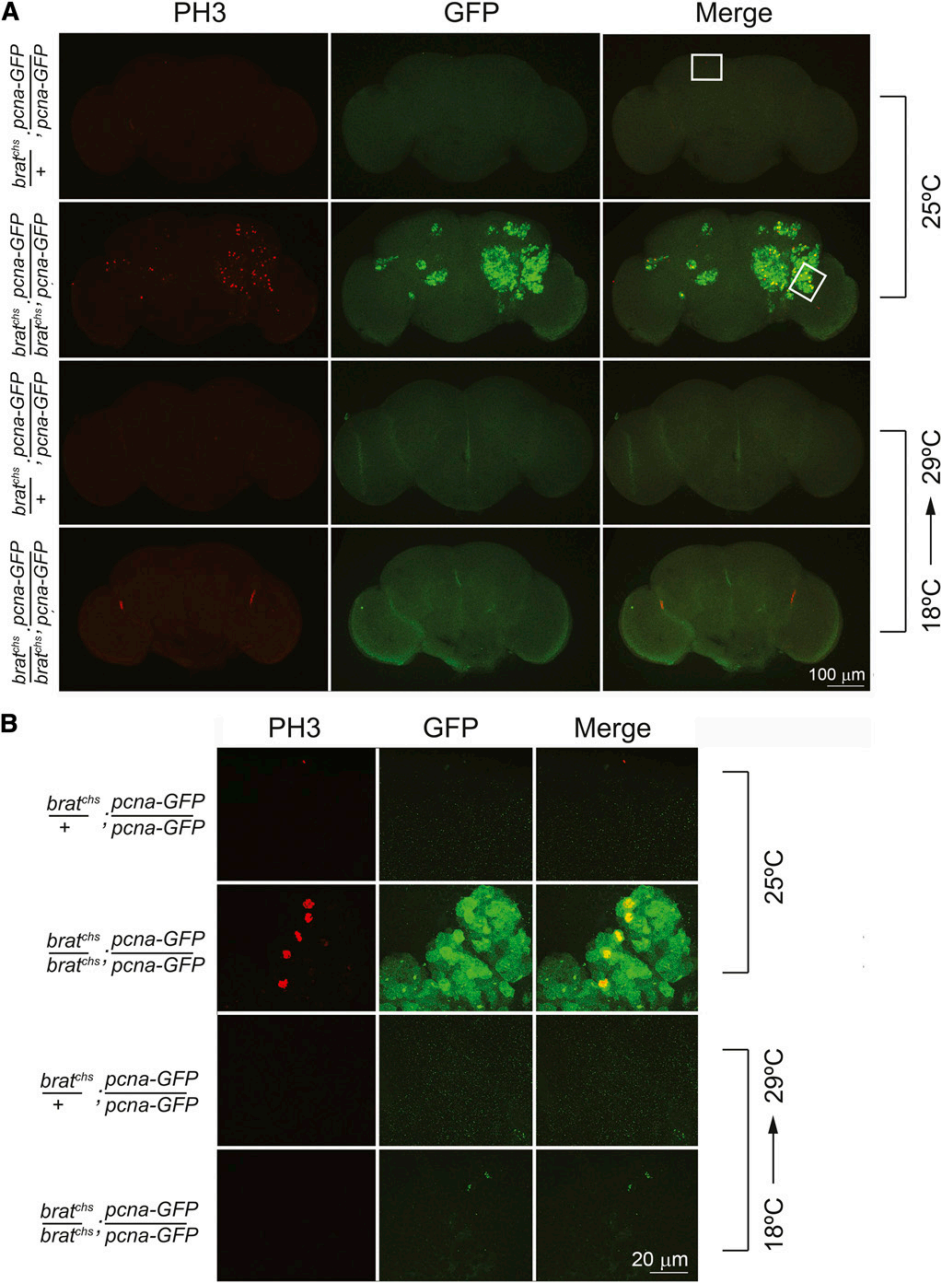
Tumor formation in *brat^chs^* adult brains entails ongoing cell division and is sensitive to temperature. A. Confocal microscope analysis of brains from *brat^chs^* mutants and control flies stained with anti-PH3 (red) and anti-GFP antibodies (green) to assay markers of active cell division. Brains are from 8-10 day old adults. *brat^chs^/+*; *pcna-GFP/pcna-GFP* (control) and *brat^chs^/brat^chs^*; *pcna-GFP/pcna-GFP* (mutant) flies were raised at 25°C (top two rows; control: n = 12 (8 females + 4 males); mutant: n = 12 (9 females + 3 males)), or at 18°C and subsequently shifted to 29°C at eclosion (bottom two rows; control: n = 6 (5 females + 1 male); mutant: n = 7 (4 females + 3 males)). Few or no mitotically dividing cells are observed in brains of *brat^chs^* adults raised at 18°C even when the flies are shifted to 29°C after eclosion. B. High magnification images of panels in A.

**Figure 6:**
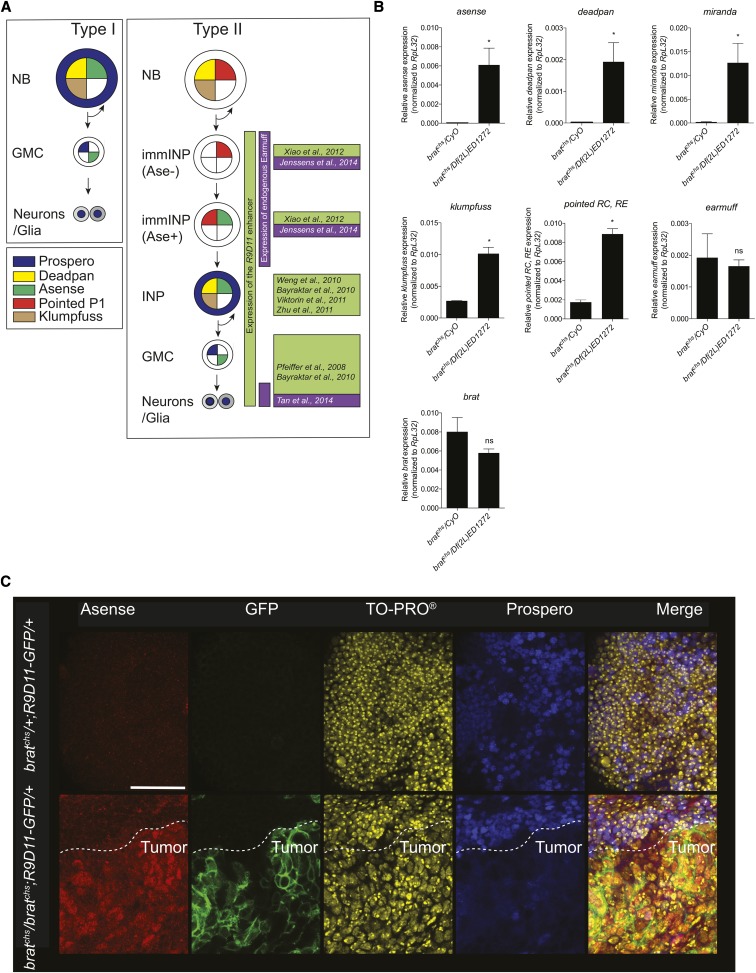
Gene expression in *brat^chs^* heads is consistent with persistent neurogenesis. A. Schematic representation of the Type I and Type II neuroblast lineages. NB: neuroblast, GMC: Ganglion Mother Cell, immINP: immature Intermediate Neural Progenitor, INP: Intermediate Neural Progenitor. Note that *pointed P1* corresponds to the *RC* and *RE* transcripts, which are specific to the Type II lineage. B. Gene expression analysis of neuroblast-specific genes in heads of *brat^chs^/CyO* (controls) and *brat^chs^/Df(2L)ED1272* adults. qPCR data showing significant upregulation of mRNA levels from the neural precursor genes *asense*, *deadpan*, *miranda*, *klumpfuss* and *pointed* isoforms *RC* and *RE* (together referred to as *pointed P1*), but not *brat* and *earmuff* mRNA levels in *brat^chs^/Df(2L)ED1272* adults. Values shown are mean ± SEM ns: not significant, *P < 0.05 based on Mann Whitney’s *U*-test. C. Confocal microscope images of brains from *brat^chs^/+*; *R9D11-mCD8-GFP /+* (controls) and *brat^chs^/ brat^chs^*; *R9D11-mCD8-GFP/+* flies stained for Asense (red), GFP (green) and Prospero (blue). Nuclei of cells are stained with TO-PRO (yellow). The brains of *brat^chs^/ brat^chs^*; *R9D11-mCD8-GFP/+* flies show increased staining for Asense and GFP in comparison with controls. In the zone of tumor formation in *brat^chs^* heads (below the dotted line), Prospero is predominantly cytoplasmic. Scale bar: 25μm.

To explore the source of the supernumerary cells in *brat^chs^* brains, we assayed these brains immunohistochemically for aberrant cell proliferation. This assay employed a *proliferating cell nuclear antigen* (*pcna)-GFP* reporter transgene that is specifically expressed in mitotic cells (Thacker *et al.* 2003). Whole brains from *brat^chs^/brat^chs^*; *pcna-GFP/pcna-GFP* and *brat^chs^/+*; *pcna-GFP/pcna-GFP* flies were stained with anti-GFP as well as with antibodies against a mitotic marker, anti-phospho-histone 3 (PH3). Consistent with published observations that there is little cell proliferation in brains of wild-type adults (Von Trotha *et al.* 2009), heterozygous control animals had no detectable *GFP* expression and few cells positive for PH3 (Figure 5A, B). In contrast, homozygous *brat^chs^* brains contained patches of cells that expressed *GFP*, some of which were actively dividing, as they also were positive for PH3 (Figure 5A and B). Hereafter, we refer to these mitotically active domains as ‘tumor regions’. In addition, DAPI staining revealed that many cells in a tumor region have abnormally large nuclei, suggesting they may be a higher than 2N DNA content (Figure S3; see also TO-PRO staining in Figure 6C and DAPI staining in Figures 3A and B).

### Neural progenitor markers are upregulated in *brat^chs^*

Many adult *Drosophila* neurons and glia in the central brain arise post-embryonically from Type I and Type II neuroblasts that undergo multiple cell divisions during larval and pupal stages (Homem and Knoblich 2012; Kang and Reichert 2015). Type I and Type II neuroblasts and their respective markers are represented in Figure 6A. On the basis of previous observations that mutations in *brat* lead to the production of supernumerary neural progenitors (Homem and Knoblich 2012; Kang and Reichert 2015), we hypothesized that the over-proliferation we observed in homozygous *brat^chs^* brains was due to abnormal proliferation of neural progenitors.

To test this hypothesis, we further examined *brat* mutants by quantitative real-time polymerase chain reaction (qPCR) on heads (Figure 6B) and immunohistochemistry on adult brains (Figure 6C). Consistent with our hypothesis, mRNA levels of four genes expressed in Type I and Type II neural progenitors: *asense* (*ase*), *deadpan* (*dpn*), *miranda* (*mira*) and *klumpfuss* (*klu*), were all upregulated in heads from hemizygous *brat^chs^/Df(2L)ED1272* flies, compared with heads from *brat^chs^/+* controls (Figure 6B). These data are consistent with a previous study that found an increase in *dpn*, *mira* and *klu* mRNA in another *brat* mutant (Loop *et al.* 2004). We also found an increase in *pointed P1* mRNA levels in *brat^chs^/Df(2L)ED1272* flies (Figure 6B), which specifically implicates involvement of the Type II lineage. We did not, however, observe increased *earmuff* (*erm*) mRNA levels (Figure 6B), which was surprising because *erm* expression is enriched in the Type II lineage (Eroglu *et al.* 2014). The previously reported expression patterns of the *erm* promoter *R9D11* and Erm protein are indicated in Figure 6A and references therein (Pfeiffer *et al.* 2008; Bayraktar *et al.* 2010; Weng *et al.* 2010; Viktorin *et al.* 2011; Zhu *et al.* 2011; Xiao *et al.* 2012; Janssens *et al.* 2014; Tan *et al.* 2015). Finally, we found that *brat* mRNA levels were not significantly reduced in *brat^chs^/Df(2L)ED1272* flies.

Immunohistochemical staining of whole brains from *brat^chs^* adults revealed tumors consisting of patches of cells with abnormal nuclei and aberrant expression of Asense, Prospero, and R9D11-mCD8-GFP that were not observed in heterozygous controls (Figure 6C). Specifically, and consistent with DAPI staining in Figures 3 and S3, TO-PRO staining revealed many cells in tumors with abnormally large nuclei (Figure 6C). Consistent with our qPCR data, Figure 6 illustrates that there is substantial Asense protein expression in the tumor region in *brat^chs^* homozygotes but not in heterozygous controls. In addition, the transcription factor Prospero localizes to nuclei in both heterozygous controls and in the non-tumor regions of homozygous *brat^chs^* brains. However, Prospero labeling is weaker and not nuclear in *brat^chs^* tumors (Figure 6C). We note that in control brains, Prospero is not uniformly expressed in neurons (Figure 6C). This non-uniformity may reflect differences in neuronal age and/or degradation of the Prospero protein in some neuronal subtypes (Vaessin *et al.* 1991; Bi *et al.* 2003). Finally, in contrast to our qPCR data, which shows no increase in *erm* mRNA (Figure 6B), we observe high levels of GFP expression from an *erm* reporter transgene (*R9D11-mCD8-GFP*) in tumor regions in homozygous *brat^chs^* brains (Figure 6C). GFP expression from this reporter is not detectable in heterozygous controls. The discrepancy between the qPCR and immunohistochemistry data for *erm* is somewhat puzzling. To confirm that *erm* expression is not upregulated in *brat^chs^/ Df(2L)ED1272* heads, we repeated the qPCR experiments with two additional sets of primers and another *brat^chs^* genotype and control (Figure S4). These experiments confirmed our original result, suggesting that the lack of *erm* mRNA upregulation in *brat^chs^/Df(2L)ED1272* heads is because immature INPs that express *erm* mRNA exist only transiently (Janssens *et al.* 2014) and rapidly revert to NBs or mature INPs that do not express *erm* mRNA. Along this line of reasoning, the GFP observed in homozygous *brat^chs^* tumors from *erm* reporter transgene expression could be due to the perdurance of GFP from immature INPs into mature INPs. This explanation is consistent with the observed weak Prospero labeling in Ase+, GFP+ cells, as immature INPs do not show Prospero expression at all. Altogether, our data lead us to conclude that the tumors in *brat^chs^* mutants contain neural progenitor cells.

### Tumor cells in *brat^chs^* can differentiate Into neurons and glia

The observation of both cellular proliferation and neurodegeneration in *brat^chs^* brains raises the question of whether the supernumerary cells are all destined to die or if some survive and differentiate. Elevated levels of GFP expression under the control of both pan-neuronal and pan-glial drivers in *brat^chs^* brains compared with controls (Figure 3) indicate that at least some of these cells persist. Furthermore, neuronal GFP staining in low magnification images shows that the mushroom bodies (MB; Figure 3A) are misplaced in homozygous *brat^chs^* flies, likely due to the presence of extra, ectopic neuropil structures (Figure 3A, arrows). This is consistent with brain sections that clearly show the presence of extra, ectopic neuropils in homozygous *brat^chs^* mutants (Figure 1D). Together these data suggest that at least some tumor cells differentiate into cells with projections, and that some of these cells express neuronal or glial markers. Consistent with these data, some *brat^chs^* brain cells expressing the *R9D11-mCD8-GFP* reporter gene make axonal-like projections that can fasciculate to form neuropil-like structures (Figure S5A). Moreover, some of these *R9D11-mCD8-GFP* expressing cells are also positive for the neuronal marker Elav, and Elav-positive cells are observed in tumor regions (Figure S5B, arrowheads in Box 1). *brat^chs^* thus is different from previously described *brat* alleles because surplus differentiated cells are produced as well as surplus neural progenitors.

### *brat^chs^* is temperature sensitive

**Figure 7:**
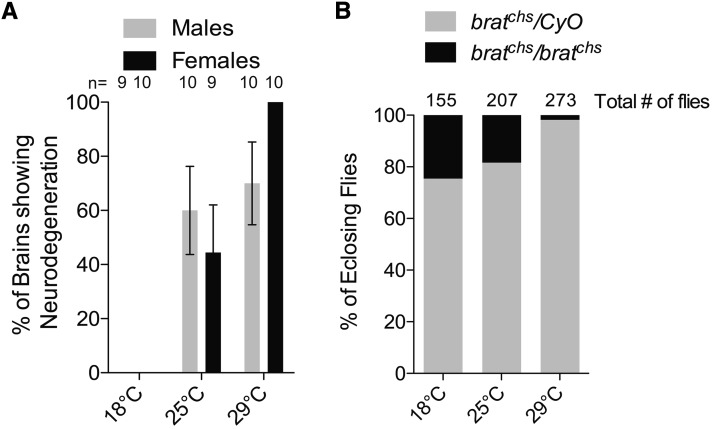
*brat^chs^* mutants are temperature-sensitive for neurodegeneration and survival to eclosion. A. The percent of *brat^chs^* adults exhibiting neurodegeneration increases with elevated temperature. Neurodegeneration is not observed in brains of 2-4 day old *brat^chs^* adults raised at 18°C (males: n = 9, females: n = 10). However, when *brat^chs^* adults are raised at 25°C (males: n = 10, females: n = 9) or 29°C (males: n = 10, females: n = 10) the fraction of flies with neurodegeneration increases up to 100% of *brat^chs^* females at 29°C. Chi-squared test: 18°C *vs.* 25°C, *P* = 0.004 for males and *P* = 0.002 for females; 25°C *vs.* 28°C: *P* = 0.06 for males and *P* = 0.001 for females. B. The percent of flies of the indicated genotypes that eclose from an intercross of *brat^chs^/CyO* males and females. Because the *CyO* balancer is lethal when homozygous, the Mendelian expectation of *brat^chs^* homozygotes among adult progeny is 33%. When progeny are raised at 18°C (n = 155) the frequency of *brat^chs^* homozygotes among the offspring approaches the Mendelian expectation but departs substantially from this value when the flies are raised at 25°C (n = 207) or 29°C (n = 273) indicating that an increasing fraction of *brat^chs^* homozygotes die before eclosion at elevated temperatures.

During the course of our experiments, we discovered that *brat^chs^* is a temperature-sensitive allele. Homozygous *brat^chs^* flies reared and aged for 2-4 days at 18° did not show any neurodegeneration, whereas the phenotype was partially penetrant (∼60% in males and ∼40% in females) for flies reared and aged for 2-4 days at 25° and more penetrant (∼70% in males and 100% in females) for flies reared and aged for 2-4 days at 29° (Figure 7A). The over-proliferation phenotype also was temperature sensitive. Brains of *brat^chs^*; *pcna-GFP* flies reared at 18° and then shifted to 29° post-eclosion, had no tumors even when aged to 8-10 days, while *brat^chs^*; *pcna-GFP* flies reared to adults at 25° do exhibit over-proliferation (Figures 5A and B). This indicates that the onset of over-proliferation is prior to eclosion and that *brat* function either is not required in the brain post-eclosion or that post-eclosion *brat* function is not affected by the *brat^chs^* allele. Consistent with the temperature-sensitivity of the over-proliferation in *brat^chs^* mutants, the eclosion rate was also temperature-sensitive. From an intercross of *brat^chs^/CyO* males and females, we expect 33% *brat^chs^* homozygotes among adult progeny because the *CyO* balancer is lethal when homozygous. At 18°, we observe an eclosion rate of 25%; at 25°, the eclosion rate drops to 18%; while at 29°, the eclosion rate is 2% (Figure 7B). Altogether, these data suggest that *brat^chs^* is a hypomorphic allele, whose function progressively declines with increasing temperature.

### The onset of over-proliferation in *brat^chs^* is prior to eclosion

**Figure 8:**
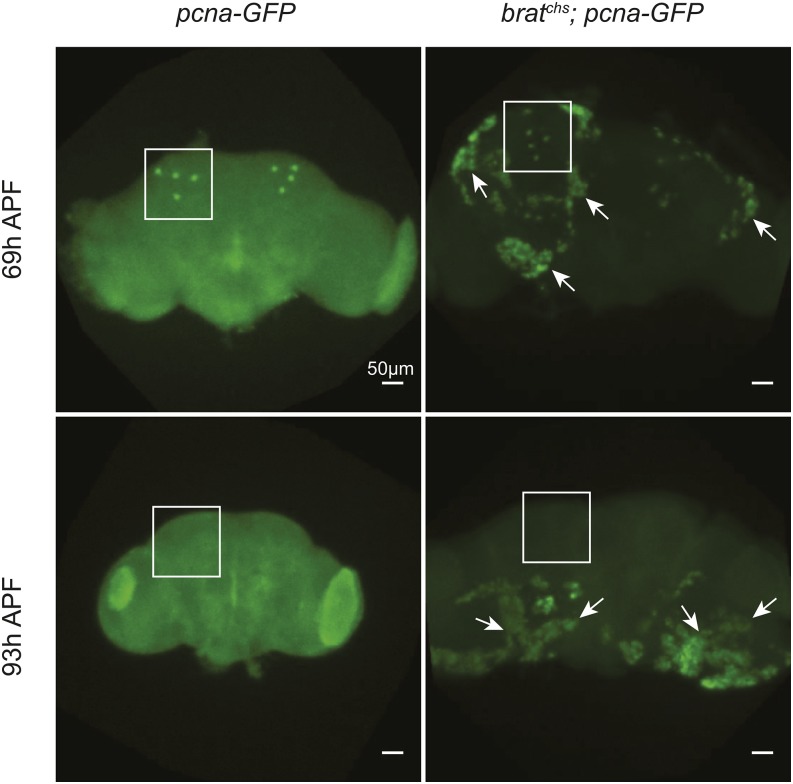
The onset of the *brat^chs^* phenotype is prior to eclosion. Representative confocal microscope images of *pcna-GFP* (control) and *brat^chs^*; *pcna-GFP* brains from animals reared at 25°C and stained for GFP (green) at 69h and 93h after puparium formation (APF). The abundance of *pcna*-expressing cells in *brat^chs^* (n = 8) but not controls (n = 4) demonstrates that the adult phenotype of *brat^chs^* is initiated by 69h APF. The four mushroom body neuroblasts per brain hemisphere (outlined by white squares) are present in both *pcna-GFP* and *brat^chs^*; *pcna-GFP* brains at 69h and disappear in *brat^chs^* by 93h APF (n = 18), as in controls (n = 17), indicating that Type I mushroom body neuroblasts are not the source of proliferating cells in *brat^chs^* adult brains.

The finding that *brat^chs^*; *pcna-GFP* flies reared at 18° until eclosion and then shifted to 29° post-eclosion exhibit neither overgrowth nor degeneration, even when the adults were maintained at 29° for an extended time (Figure 5 and data not shown), suggests that the critical period for *brat* activity is prior to eclosion. To further assess when brain tumor formation is initiated in *brat^chs^*, we again assayed for cell proliferation using the *pcna-GFP* transgene. Supernumerary proliferating cells are present in the brain of *brat^chs^/brat^chs^*; *pcna-GFP/pcna-GFP* pupae at 69 hr after puparium formation (APF) at 25° (Figure 8, arrows). Supernumerary proliferating cells are still evident in *brat^chs^/brat^chs^*; *pcna-GFP/pcna-GFP* pupae at 93 h APF at 25° (Figure 8, arrows). Because the four mushroom body neuroblasts in each brain hemisphere are the last to become mitotically quiescent during development (Siegrist *et al.* 2010), we asked whether persistence of these neuroblasts could account for over-proliferation in *brat^chs^* mutants. However, the mushroom body neuroblasts appear to become quiescent on schedule; they were no longer detected in *brat^chs^/brat^chs^*; *pcna-GFP/pcna-GFP* brains by 93 hr APF (Figure 8, boxed regions). While we have not ruled out the possibility that the mushroom body neuroblasts persist, but in altered locations, these results confirm that over-proliferation in *brat^chs^* flies is initiated prior to eclosion. Together with the immunohistochemistry and qPCR data presented above, we think it is likely that the proliferating cells in *brat^chs^* adults are of the Type II lineage.

### *CG15864* is an enhancer of *brat^chs^* and encodes a putative prolyl 4-hydroxylase

**Figure 9:**
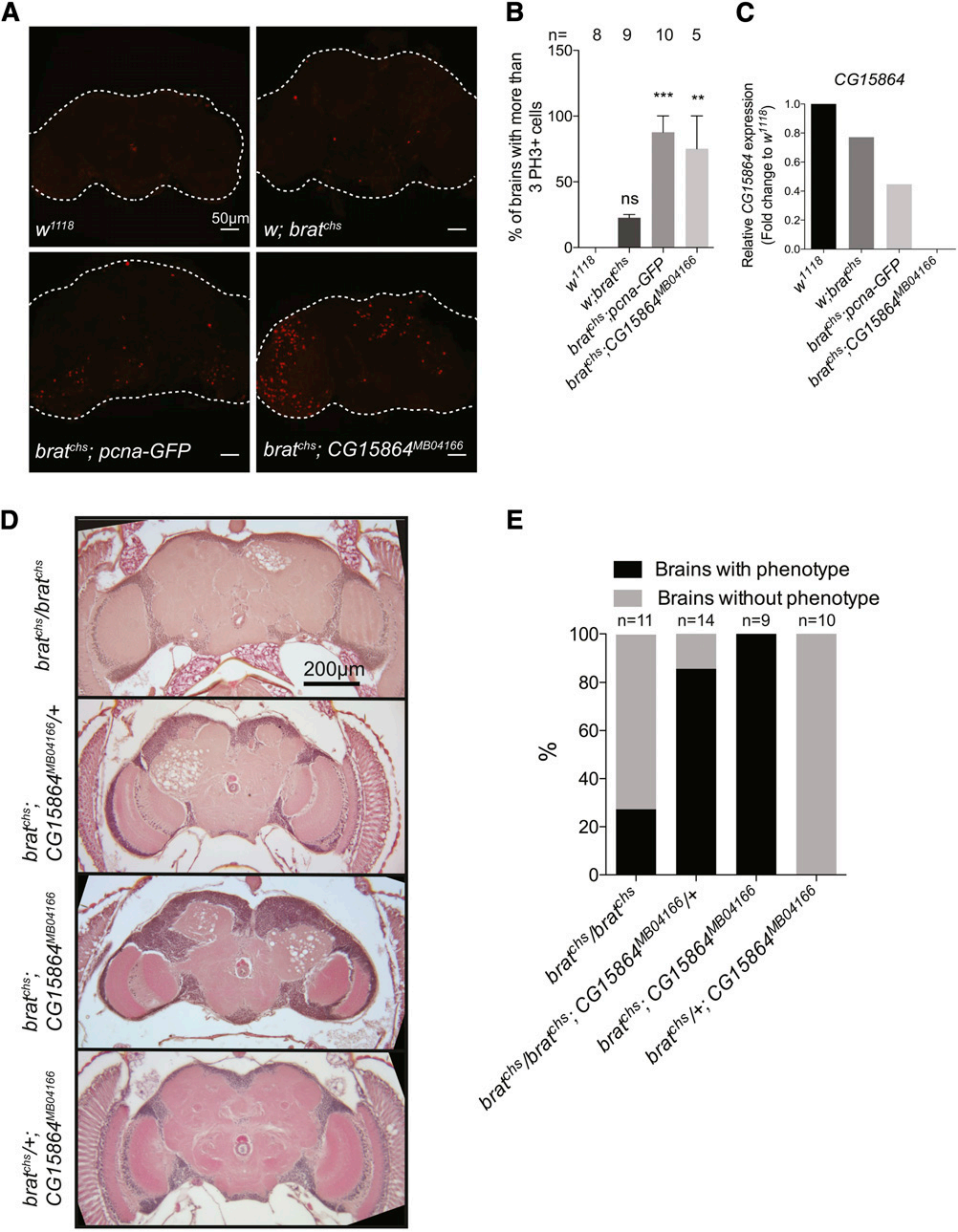
*CG15864* is a modifier of *brat*. A. Representative confocal microscope images of whole brains from: *w^1118^*, *w*; *brat^chs^*, *brat^chs^*; *pcna-GFP*, and *brat^chs^*; *CG15864 ^MB04166^* 1-4 adult brains stained with the mitotic marker PH3 (red). B. Quantification of cell proliferation in the brains of flies of the indicated genotypes as the percentage of brains having more than 3 PH3-positive cells. Significance was calculated using one-way ANOVA test. **P < 0.001 and ***P < 0.0001. C. qPCR analysis of *CG15864* mRNA in heads of flies of the indicated genotypes. Values represent the fold-change in *CG15864* expression compared with *w^1118^* controls. At least three biological replicates were used for each genotype. D. Representative 5-μm paraffin sections of adult brains from w; *brat^chs^*, *brat^chs^*; *CG15864^MB0416^/+*, *brat^chs^*; *CG15864^MB04166^* and *brat^chs^/+*; *CG15864^MB04166^* flies. E. Quantification of neurodegeneration phenotypes observed by histology in D. For this graph, brains were scored as having or lacking neurodegeneration. The severity of the pathology was not scored here. Chi squared test: *P* < 0.001.

In previous experiments utilizing the *pcna-GFP* reporter, we noticed that *brat^chs^* flies carrying this reporter exhibited more severe neurodegeneration (compare Figure 1A and Figure 2) and over-proliferation (Figures 9A and B) than *brat^chs^* flies lacking the reporter. This raised the possibility that the *pcna-GFP* insertion disrupted the function of a gene that interacts with *brat*. We mapped the *pcna-GFP* transgene insertion using splinkerette PCR (splinkPCR; (Potter and Luo 2010)) to the *CG15864* locus at position 1724 of the gene in an intronic region. *CG15864* encodes a putative prolyl 4-hydroxylase orthologous to human *prolyl 4-hydroxylase subunit alpha 1*, *3*, *2 and TM* (*P4HA1*, *P4HA3*, *P4HA2 and P4HTM*). Measurement of *CG15864* mRNA levels by qPCR in heads from various genotypes including one containing another transposon insertion in *CG15864* (*CG15864^MB04166^*) revealed that *CG15864* mRNA levels are reduced ∼20% in *brat^chs^*/*brat^chs^* heads, compared with controls, and that *CG15864* transcript levels are reduced an additional ∼40% in *brat^chs^*; *pcna-GFP* heads (Figure 9C). *CG15864* mRNA levels in *brat^chs^*; *CG15864^MB04166^* heads were almost undetectable. These data confirm that the *pcna-GFP* insertion disrupts the *CG15864* gene. Furthermore, Brat apparently regulates the steady-state level of *CG15864* mRNA in adult heads. The difference in expression of *CG15864* between *brat^chs^* mutants that carry *pcna-GFP* and those that carry *CG15864^MB04166^* is most likely because the *pcna-GFP* transgene is inserted into an intron of *CG15864* while *CG15864^MB04166^* contains a *P*-element insertion in an exon of *CG15864*.

If the decrement in *CG15864* expression associated with the *pcna-GFP* insertion were responsible for enhancement of *brat^chs^* phenotypes, we would expect *CG15864^MB04166^* to cause similar enhancement. We tested this prediction by asking whether *CG15864^MB04166^*, like *pcna-GFP*, enhanced over-proliferation and neurodegeneration in *brat^chs^*. Both *brat^chs^*; *pcna-GFP* and *brat^chs^*; *CG15864^MB04166^* brains, exhibit higher numbers of PH3 positive cells compared with *w^1118^* controls or *brat^chs^* mutants in the absence of a *CG15864* mutation (Figures 9A and B). Similarly, in comparison with *brat^chs^/+*; *CG15864^MB04166^* and *brat^chs^* flies, neurodegeneration in *brat^chs^*; *CG15864^MB04166^* brains is 100% penetrant (Figures 9D and E) as in *brat^chs^*; *pcna-GFP* brains (Figure 2). Additionally, one copy of *CG15864^MB04166^* is sufficient to enhance neurodegeneration in *brat^chs^* flies (Figures 9D and E). Together, these data indicate that *CG15864* interacts with *brat* to prevent both over-proliferation and neurodegeneration.

## Discussion

### A novel *brat* allele

Here we describe *cheesehead*, a novel mutation of *brain tumor* in *Drosophila*. *Drosophila* Brat is a TRIM-NHL protein composed of two B-Box domains, a Coiled-Coil domain, and an NHL domain. The NHL and Coiled-Coil domains of Brat are essential for its interaction with the adaptor protein Miranda (Komori *et al.* 2014). This interaction localizes Brat to the basal side of proliferating neuroblasts such that during asymmetric cell division of Type II neuroblasts, Brat becomes segregated into the smaller, non-neuroblast daughter cell where it then specifies the immature INP identity through a mechanism involving the B-Boxes (Lee *et al.* 2006; Komori *et al.* 2014). Whereas most reported *brat* alleles have mutations in the NHL domain (Arama *et al.* 2000), *cheesehead* is temperature-sensitive mutation in the Coiled-Coil domain of the protein.

Temperature-sensitive phenotypes typically are thought to result from changes in protein folding when synthesis occurs at the restrictive temperature (Edgar and Lielausis 1964; Gordon and King 1994) or from protein destabilization and loss of protein function when shifted to the restrictive temperature (Sadler and Novick 1965). Destabilizing mutations can be found in nucleic acid-binding domains or protein-protein interaction domains affecting protein structure such that proteins become misfolded and/or can no longer interact appropriately with essential binding partners at restrictive temperatures (Lovato *et al.* 2009). In the case of *brat^ch^*^s^ it is possible that disruption of an essential cofactor or target interaction mediated by the Coiled-Coil domain at higher temperatures leads to the observed phenotypes. Given that the Coiled-Coil domain is required for Brat’s interaction with Miranda and, thus its proper segregation into the non-neuroblast daughter cell during neuroblast division, Brat may be incorrectly partitioned at restrictive temperatures in *brat^chs^* mutants. This would lead to daughter cells with insufficient amounts of Brat to progress through differentiation. In this case, the Brat deficient cells might revert to a more neuroblast-like fate and continue proliferating, giving rise to tumors. Indeed, previously reported *brat* mutations lead to the formation of supernumerary neuroblasts at the expense of differentiated cells (Lee *et al.* 2006), leading to enlarged larval brains and most often to lethality prior eclosion (Arama *et al.* 2000). However, in contrast to most of these previously reported mutations, *brat^chs^* mutants survive into adulthood and develop brain tumors that contain supernumerary differentiated cells, as well as neuronal precursors. These observations suggest that the decision to maintain or revert to neural stem cell fate or to differentiate may be stochastic in *brat^chs^* mutants.

We attempted to examine the neurodegeneration phenotype of other, weaker *brat* alleles, that were also reported to be adult viable (*e.g.*, *brat^k06028^*, *brat^ts1^* and *brat^fs1^*) (Arama *et al.* 2000). An adult brain overgrowth phenotype had been observed in homozygous *brat^k06028^* mutants carrying a transposable element inserted into a non-coding region of the *brat* locus (Loop *et al.* 2004). However, we were unable to obtain viable adults even at low temperatures (18°) from any of the abovementioned lines and therefore couldn’t assess their neurodegenerative phenotypes. Nonetheless, the fact that neural progenitor-specific knock down of *brat*, as well as *NICD* overexpression leads to neurodegeneration, recapitulating the spongiform pathology observed in *brat^chs^* flies, supports the idea that Brat plays a neuroprotective role. More work is needed to establish how exactly the CC domain contributes to this process and whether the B-Box- and NHL- domains are also critical for Brat’s neuroprotective activity.

The current prevailing view, based on analysis of larval brains, is that *brat* functions primarily in the proliferation of Type II lineages (Sonoda and Wharton 2001; Betschinger *et al.* 2006; Bello *et al.* 2008; Bowman *et al.* 2008). We performed qPCR and immunohistochemical analyses to investigate the identity and origins of the proliferating cells in *brat^chs^* adult brains. The upregulation of *ase* mRNA (in *brat^chs^/Df(2L)ED1272* heads) and protein (in homozygous *brat^chs^* brain tumors) could reflect the presence of Ase+ immature INPs, mature INPs and/or GMCs from the Type II lineage, consistent with the idea that the Type II lineage is affected by *brat^chs^* in adult brains. Additionally, *pnt P1* mRNA, which is thought to be specific to the Type II lineage, also is upregulated in *brat^chs^/Df(2L)ED1272* heads. Although mRNA levels of *erm*, which is also present in Type II progenitor cells, were not elevated in *brat^chs^/Df(2L)ED1272* heads, we did find *GFP* expression from an *erm* reporter transgene in homozygous *brat^chs^* tumors. Together with our finding that Type I mushroom body neuroblasts in homozygous *brat^chs^*mutants become mitotically quiescent on schedule, we think it is unlikely that the Type I lineage is affected in *brat^chs^* brains. The fact that high levels of Ase are observed in the tumors of these flies, suggests that the majority of proliferating cells are not Type II neuroblasts, but rather more differentiated progeny (such as INPs) with the ability both to self-renew and give rise to differentiated neurons and glia.

### Caspase activation in *brat^chs^* brains

The most intriguing aspect of *brat^chs^* is its dual brain phenotype. *brat^chs^* brains exhibit both abnormal cell proliferation resulting in overgrown brains and degeneration resulting in a spongiform pathology of the central brain. Our observation that *brat^chs^* brains are positive for activated Dcp-1 suggests that programmed cell death is associated with tissue loss. The presence of apoptotic cells has been established in several human tumor types and, in some cases, has been positively correlated with tumor severity. However, the relationship between apoptotic cells and tumor formation and progression is still unclear (Jäger and Zwacka 2010).

We identified two classes of cells that are positive for cleaved Dcp-1. The first class comprises about half of each tumor region, and these cells are weakly Dcp-1 positive. Cells in the second class, which are rare, are strongly positive for cleaved Dcp-1. This finding may be analogous to results with human oral squamous cell carcinomas, where weak activation of human Caspase-3 is observed in proliferative cells, and strong activation of Caspase-3 is observed in apoptotic cells within the same tumors (Heshiki *et al.* 2015). Thus, we hypothesize that the cells weakly positive for cleaved Dcp-1 are proliferating, whereas the cells strongly positive for cleaved Dcp-1 are undergoing apoptosis. Consistent with this hypothesis, the nuclei of strongly Dcp-1-positive cells are often not visible or pyknotic. Importantly, caspases have been implicated in non-apoptotic roles that may be relevant to tumor formation and progression such as cellular proliferation and differentiation (Jäger and Zwacka 2010). For example, Caspase-3 has been implicated in the differentiation of murine neuronal stem cells (Fernando *et al.* 2005) through its cleavage of the stemness factor Nanog (Fujita *et al.* 2008). Although cleaved Dcp-1 has not yet been shown to play this type of non-apoptotic role in *Drosophila*, it could explain the weak cleaved Dcp-1 signal in *brat^chs^* brain tumors.

One potential explanation for the presence of apoptotic cells in the brains of *brat^chs^* flies is that they are recapitulating the cell death that occurs during normal development. Programmed cell death in the Type II neuroblast lineage occurs during larval and pupal stages to eliminate excess neurons and assure proper neuronal connectivity and neuropil formation of the adult central complex (Jiang and Reichert 2012). Developmental death might continue into adulthood in *brat^chs^* because of the ongoing proliferation and differentiation of ectopic progenitor cells. The fact that some of the apoptotic cells in *brat^chs^* brains are positive for GFP from the *erm* reporter transgene favors this explanation, because during normal development some cells in the larval and pupal brain that express GFP from a similar *erm* reporter construct were also strongly positive for activated Caspase-3 (Jiang and Reichert 2012). Another possibility is that the apoptotic cell death that we see in *brat^chs^* flies is a protective mechanism against the tumorous overgrowth and that Dcp-1 plays a tumor suppressor role in this context. This would be consistent with the well-documented roles of caspases as tumor suppressors (reviewed in (Olsson and Zhivotovsky 2011)).

None of the apoptotic cells in *brat^chs^* brains express the neuronal gene *nSyb*. Only a few dying cells express the glial gene *repo* while most express the neural progenitor gene *erm*. While it is possible that some of the dying cells have lost glial and/or neuronal gene expression, our data suggest that differentiated neurons and glia are not the main cell type undergoing apoptotic cell death in homozygous *brat^chs^* brains. This was surprising to us, because the holes we observe histologically are in neuropil, which consists of glial cells and the axons and dendrites of neurons. Thus, the relationship of the apoptotic cells in *brat^chs^* tumors to the holes present in histological preparations of *brat^chs^* brains is puzzling. We offer three potential explanations. The first is that the holes represent areas of axonal retraction and are independent of apoptosis. A second possibility is that the degenerating areas of neuropil in *brat^chs^* brains represent processes of dying tumor cells that do not express or have lost canonical glial and neuronal markers. A third possibility is that glia and/or neurons are dying in *brat^chs^* mutants, but via non-apoptotic mechanisms.

The first possibility stems from our observation that when ectopic neuropil is clearly visible in histological preparations it is usually full of holes (*e.g.*, Figure 1D). Therefore, it may be that degenerating neuropil in *brat^chs^* represents degenerating projections from tumor cells. These projections could degenerate either because they are inappropriately targeted or because they are not properly maintained. It recently was reported that Brat is critical for maintenance of mushroom body axons via repression of Src64B kinase (Marchetti *et al.* 2014), which is a key player in the Rho-dependent genetic pathway that controls axon retraction (Billuart *et al.* 2001). It could be that Brat plays a broader role in axonal maintenance and that the degenerating neuropil in *brat^chs^* is due to excessive axonal retraction. We tested this by comparing immunohistochemistry and histology data from brains of *brat^chs^* flies and brains in which *brat* activity was reduced in mushroom bodies (*OK107-Gal4 > UAS-mCD8-GFP*, *UAS-brat_RNAi_*). Using Fas II antibody we labeled the mushroom bodies and observed axon retraction (Figure S6). This is consistent with previous findings (Marchetti *et al.* 2014). However, by histology, we did not detect spongiform pathology in or near the mushroom bodies (Figure S7). Moreover, Fas II staining in *brat^chs^* flies was comparable to controls (Figure S6). We therefore do not think axon retraction accounts for the spongiform pathology observed in *brat^chs^* brains.

A second possibility is that the tumor cells die, giving rise to holes in the neuropil, because they do not express the correct sets of genes normally active in differentiated neurons or glia. For example, *erm* expression should be limited to neural progenitors in the central brain and a few differentiated neurons in the optic lobes (Tan *et al.* 2015). However, in *brat^chs^* central brains, some cells expressing the *erm* reporter transgene send out projections and therefore morphologically resemble neurons rather than neural progenitors. If cells initiate differentiation without down-regulating stem cell genes, the lack of a clear cell identity could trigger cell death. Loss of such cells and their projections could underlie the neuropil holes in homozygous *brat^chs^* mutants.

A third possibility is that non-apoptotic cell death contributes to the spongiform pathology we observe in *brat^chs^* mutant brains. Non-apoptotic death, including necrosis, necroptosis and autophagy, has been reported in mammalian neurodegeneration following ischemic injury and in certain neurodegenerative diseases (Troulinaki and Tavernarakis 2012; Probert 2015; Clark and Vissel 2016). Dcp-1 is not activated in some non-apoptotic cell death. It therefore is possible that dying glia and/or neurons not detected in our experiments account for the observed pathology in *brat^chs^* mutant brains. We note that these three possibilities are not mutually exclusive and that a combination of axon retraction and multiple types of cell death may be responsible for the neurodegeneration phenotype we observe in *brat^chs^* mutants.

### *CG15864* as a modifier of the *brat^chs^* phenotype

We found that *brat^chs^* phenotypes are enhanced by elevated temperature and by a mutation in an uncharacterized gene that likely encodes a prolyl4-hydroxylase. Sequence analysis of *CG15864* indicates that the encoded protein is characterized by four domains, including an Oxoglutarate/iron-dependent dioxygenase, a Tetratricopeptide-like helical domain, a Prolyl 4-hydroxylase, alpha subunit and a Prolyl 4-hydroxylase alpha-subunit, N-terminal domain (see Flybase report for). *CG15864* is evolutionarily conserved. Its human orthologs encode Prolyl 4-Hydroxylase Alpha (P4HA) subunits (OMIM IDs: P4HA3: 608987, P4HA1: 176710, P4HA2: 600608) and a transmembrane Prolyl 4-Hydroxylase (P4HTM, OMIM ID: 614584). As their names imply, these enzymes add hydroxyl moieties to proline residues in other proteins. P4HA and P4HTM play roles in collagen synthesis (Gorres and Raines 2010) and regulation of Hypoxia-Inducible Factors (HIFs) (Oehme *et al.* 2002; Koivunen *et al.* 2007; Gorres and Raines 2010). In normoxic conditions, hydroxylation of proline residues by Prolyl 4-Hydroxylase targets HIF-1α for degradation by the proteasome. In hypoxic conditions HIF-1α is not hydroxylated and escapes degradation, allowing it to dimerize with HIF-1β and to activate the transcription of target genes (Chowdhury *et al.* 2008; Gorres and Raines 2010). Importantly, activation of hypoxia signaling pathways is consistently and strongly associated with aggressive malignancy (Harris 2002), and HIF-1 factors also seem to play a role in the maintenance of cancer cell stemness (Yun and Lin 2014). Thus, it may be that reducing the level of the P4H encoded by *CG15864* may enhance the *brat^chs^* phenotype by activating a hypoxia program. More work is needed to test whether *CG15864* also enhances the tumor phenotypes of other *brat* alleles, and whether the enhancement occurs via regulation of hypoxia genes or via another mechanism, such as hydroxylation of Brat itself. In either case, identification of this enhancer is likely to lead to previously unknown functions for *brat* that are likely to be relevant to human health.

In summary, we have identified a temperature-sensitive allele of the *brat* gene, *brat^chs^* that exhibits a novel dual phenotype and is likely to be highly useful for genetic dissection of *brat* function. *brat^chs^* mutants have both the characteristic over-proliferation phenotype, as well as a novel neurodegeneration phenotype. We have been unable to uncouple these seemingly disparate phenotypes. Our data do not distinguish among the following possibilities: 1) over-proliferation leading to neurodegeneration; 2) neurodegeneration leading to over-proliferation; or 3) independent processes being responsible for each of these phenotypes. An important avenue for future research is to determine whether one phenotype triggers the other, or whether the phenotypes result from perturbing distinct genetic programs. The over-proliferation phenotype in *brat^chs^* brains also appears to be somewhat different from that of previously described alleles. Like other alleles, *brat^chs^* brains exhibit over-proliferation of neuroblasts and INPs. However, unlike other alleles, *brat^chs^* brains also produce an excess of differentiated cells. We do not yet know whether this difference is due to the location of the mutation in the Coiled-coil domain or because the Brat^chs^ protein retains more wild type functionality. Distinguishing between these possibilities also will be an important area for future work. Finally, the homozygous viability and temperature sensitivity of the *brat^chs^* allele offer opportunities for genetic analysis of *brat* function that were not available previously. For instance, using this allele, it now will be possible to screen for suppression or enhancement of the adult over-proliferation and/or neurodegeneration phenotypes in adult brains to identify genes with which wild type *brat* interacts to regulate proliferation and prevent neurodegeneration. Further, by adjusting the timing of temperature shifts from permissive to non-permissive temperatures, it will be possible to identify different genes with which *brat* interacts at distinct developmental stages.

Increasing evidence also indicates that cancer and neurodegeneration share common genes and pathophysiological processes (Snyder *et al.* 2017). Mutations in genes involved in cell cycle regulation, oxidative stress and protein turnover are characteristic for both types of conditions (Morris *et al.* 2010). For example, mutations or deletions in the human E3-ubiquitin ligase-coding gene *PARK2* that lead to increased levels of cyclin E and re-entry of the cell cycle, are associated with both, several malignancies and early onset Parkinson’s disease (Morris *et al.* 2010). Indeed, recent epidemiological studies have identified positive associations between Parkinson’s disease and an increased risk of malignant brain tumors (Lin *et al.* 2015; Ye *et al.* 2016). *brat^chs^* flies may serve in the future as an excellent model to investigate the mechanisms that underlie both conditions and may open novel avenues for therapeutic strategies and more targeted treatments for both cancer and neurodegenerative disease.
